# Prenatal and Postnatal Management of Hydronephrosis

**DOI:** 10.1100/tsw.2009.85

**Published:** 2009-07-13

**Authors:** Pravin K. Rao, Jeffrey S. Palmer

**Affiliations:** ^1^ Glickman Urological and Kidney Institute, Cleveland Clinic, Cleveland, OH, USA; ^2^ Glickman Urological and Kidney Institute, Cleveland Clinic Children's Hospital, Cleveland Clinic Lerner College of Medicine of Case Western Reserve University, Cleveland, OH, USA

**Keywords:** hydronephrosis, prenatal, urology, vesicoureteral reflux, ureteropelvic junction obstruction

## Abstract

The majority of pregnant women in the U.S. undergo prenatal ultrasonography and approximately 0.5% of these examinations will detect fetal malformations. Up to one-half of these abnormalities include the genitourinary system and the most common urological finding is hydronephrosis. Some conditions associated with prenatal hydronephrosis portend a poor prognosis, while others can follow a fairly benign course. This review focuses on the definition and prenatal assessment of hydronephrosis, fetal intervention, and postnatal management.

## INTRODUCTION

Routine prenatal ultrasonography (PNUS) is usually performed at approximately 7–8 and 16–20 weeks gestation. In 1980, the exam was performed in approximately 33% of all pregnancies and in 2002, it was done for an estimated 65% of all pregnant patients[1,2]. The purpose of the routine exam includes determination of the number of pregnancies, precise calculation of gestational age, localization of the placenta, and detection of fetal anomalies. Ultrasound examinations might also be done to assess fetal health and viability in the evaluation of maternal vaginal bleeding or other worrisome events during pregnancy.

In a study of 12,000 prenatal sonograms, fetal malformations were found in 0.5% of all pregnancies and studies have suggested that 20–50% of detected structural abnormalities involve the genitourinary system[1,3]. Hydronephrosis is the most common urinary tract finding on PNUS and it can often be of little consequence to the patient. However, certain diagnoses associated with hydronephrosis or other genitourinary abnormalities can have significant bearing on postnatal outcome and fetal intervention. In fact, in a 20-year review, it was found that pregnancies were electively terminated due to spina bifida in 65%, posterior urethral valves (PUV) in 46%, prune belly syndrome in 31%, and exstrophy in 25% of cases[4]. Importantly, when pregnancy is not terminated, early detection allows for the identification of patients that might benefit from early intervention.

The rapid transmission of sound through fluids allows ultrasonography to have particularly good imaging of fluid collections and their bordering structures. The renal pelvis, the bladder, and amniotic fluid are areas of normal fluid collections that reflect the urologic system on PNUS. While normal ureters are usually not visualized on PNUS, dilated ureters can be seen with various structural and functional urological abnormalities.

The quantity of amniotic fluid is a particularly good marker of the overall health of the fetus; in fact, mid-gestational oligohydramnios is the best predictor of pulmonary hypoplasia[1]. Amniotic fluid volume can also give information about the degree of suspected urinary tract obstruction in the setting of hydronephrosis or a distended bladder. Other fluid collections that can indicate urologic abnormalities include urinoma, dilated posterior urethra, urinary tract duplication, and renal cysts. The urothelium can be assessed at its interface with urine and renal parenchymal echogenicity should be assessed as well. In any patient, when performing or interpreting PNUS, it is crucial to consider overall fetal well-being, gestational age, and unilaterality vs. bilaterality[5]. This review focuses on the definition and prenatal assessment of hydronephrosis, fetal intervention, and postnatal management.

## DIAGNOSIS OF HYDRONEPHROSIS

Multiple factors make it difficult to set a single threshold measurement to define hydronephrosis in the fetus. First, the collecting system of the kidney grows as gestation progresses normally, so no standard measure can apply to all gestational ages. Second, there is a large variety of definitions of prenatal hydronephrosis in the literature. Finally, “abnormal” can be defined in a variety of ways. For example, some possible definitions for prenatal hydronephrosis include: variation from the average, threshold of pathological or clinical significance, or likelihood of having hydronephrosis on postnatal ultrasonography.

The most commonly used measurement for prenatal hydronephrosis is the anterioposterior diameter (APD) of the renal pelvis. Many grading systems are presented with various clinical correlates. The most commonly used grading system for infants after 20 weeks gestation is as follows[6]:

- Grade 1: Renal pelvis APD 1 cm and no caliectasis (Fig. 1)
- Grade 2: Renal pelvis APD 1–1.5 cm and no caliectasis
- Grade 3: Renal pelvis APD >1.5 cm and slight caliectasis
- Grade 4: Renal pelvis APD >1.5 cm and moderate caliectasis
- Grade 5: Renal pelvis APD >1.5 cm and severe caliectasis with cortical atrophy (thickness <2 mm) (Fig. 2)

## ASSOCIATED CONDITIONS

The finding of hydronephrosis on PNUS can indicate multiple conditions. Some are associated with high morbidity, while others have a fairly benign natural course. The differential diagnosis of prenatal hydronephrosis is listed in Table 1 and is discussed in more detail below.

### Vesicoureteral Reflux

Vesicoureteral reflux (VUR) is the retrograde flow of urine from the bladder into the ureters. VUR can develop secondary to obstructions, including posterior urethral valves or a prolapsed ureterocele. However, more commonly, VUR is primary, resulting from developmental abnormalities of the ureterovesical junction (UVJ). In the absence of infection in the urine, VUR itself is thought to be benign. In general, boys are more likely to have severe, bilateral hydronephrosis from primary VUR, and they are more likely to have secondary VUR from bladder outlet obstruction.

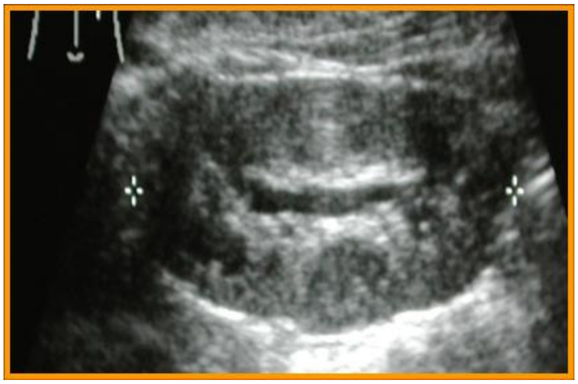

**FIGURE 1.** Grade 1 hydronephrosis on ultrasonography. Renal pelvis APD 1 cm and no caliectasis.

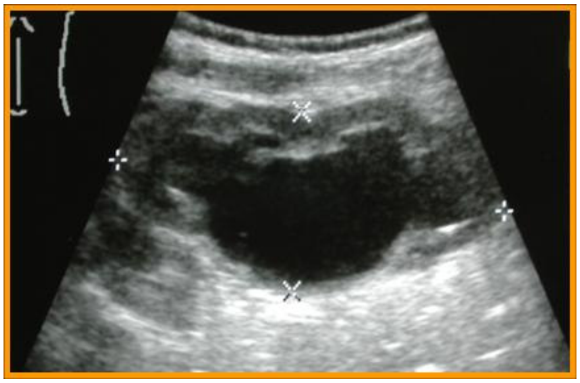

**FIGURE** 2. Grade 5 hydronephrosis on ultrasonography. Renal pelvis APD >1.5 cm and severe caliectasis with cortical atrophy (thickness <2 mm).

**TABLE 1**
**Differential Diagnosis of Prenatal Hydronephrosis**

| Condition: |
|---|
| • Vesicoureteral reflux |
| • Ureteropelvic junction obstruction |
| • Posterior urethral valves |
| • Ureterocele |
| • Primary nonrefluxing megaureter |
| • Ureterovesical junction obstruction |
| • Ureteral stricture |
| • Ectopic ureter |
| • Prune-belly syndrome |

While patients with high-grade reflux are more likely to have had more severe hydronephrosis, approximately 10–30% of patients with prenatal hydronephrosis and 30–35% of patients with severe prenatal hydronephrosis will have primary VUR[5,7,8]. Thus, it is not possible to predict reflux or its grade based on prenatal imaging. Moreover, while VUR can appear as hydronephrosis on PNUS, there are no specific signs to indicate its presence.

Studies of patients with prenatal hydronephrosis due to VUR showed that reflux resolved or improved by 4 years of age in around 75% of cases, without any detected anatomical or physiological effects[9,10]. However, due to the benefit of prophylactic antibiotics for many patients, evaluation for reflux is usually performed with postnatal exams, including voiding cystourethrogram (VCUG) or radionuclide cystogram. Ironically, postnatal ultrasonography will often be normal in patients that had prenatal hydronephrosis due to VUR; in fact, one study showed that 27% of patients with prenatal hydronephrosis and grade 2–5 VUR had normal postnatal renal ultrasonography[1,11]. Prophylactic antibiotics have shown potential to reduce harmful infections in children with VUR and early circumcision of males might augment this effect. These steps can be taken prior to deciding definitive management for a given patient.

## Ureteropelvic Junction Obstruction

Ureteropelvic junction obstruction (UPJO) accounts for 44–65% of cases of prenatal hydronephrosis[12] and is unilateral in 90% of cases. It is most commonly diagnosed on PNUS, although it can present later with flank pain or urinary tract infection (UTI). In the pediatric population, UPJO is usually due to an adynamic, narrowed portion of the ureter, but the obstruction can also be due to a crossing accessory renal vessel. The latter, however, is more commonly the case in adults rather than in children.

Most commonly, hydronephrosis due to UPJO is reassessed postnatally with functional and anatomical studies, including ultrasonography, MAG-3 diuretic renogram, retrograde pyelograms, and/or captopril renography. Postnatally, UPJO can be diagnosed by CT scan and MRI (Fig. 3).

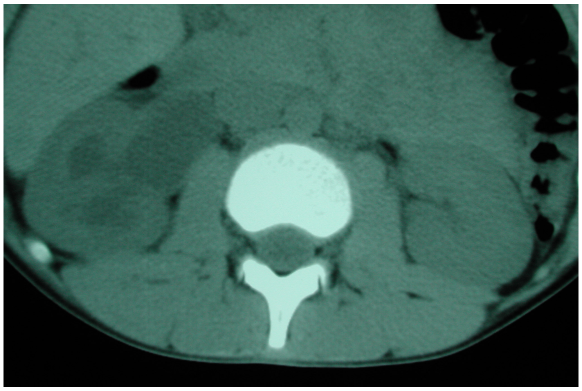

**FIGURE 3**. Ureteropelvic obstruction on CT scan. Dilatation of right renal collecting system.

While benefits to prenatal diagnosis with regards to UPJO have not been clearly delineated, a few publications have suggested that early treatment, when indicated, leads to improved renal function outcomes. Chertin et al. reported on renal function outcomes in 113 patients who had prenatal hydronephrosis and ultimately underwent pyeloplasties for UPJO. Compared to patients followed carefully from birth, the authors reported worsened preoperative renal function and decreased postoperative improvement in patients that were initially lost to follow-up before presenting later with symptoms from their obstruction[13]. In a Society for Fetal Urology randomized study, the authors found that children under 6 months of age with good renal function and unilateral obstructive hydronephrosis had better outcomes with pyeloplasty than with observation[14]. Although these findings demonstrate an indirect benefit of prenatal detection of UPJO, other studies have suggested that a high percentage of cases of severe unilateral primary hydronephrosis either resolve or improve spontaneously[15]. If this were validated, then the potential benefit of prenatal diagnosis would be controversial.

## Ureterocele

A ureterocele is a cystic dilation of the distal ureter found four times as frequently in girls than boys (Fig. 4). The PNUS can show a cystic mass in the bladder, and the kidney and ureter are often distended down to the level of the obstructing ureterocele. Eighty percent of patients with ureterocele will also have duplication of the ipsilateral collecting system, usually with a ureterocele of the upper pole ureter[16]. In addition, the upper pole will often have compromised function due to obstruction from the ureter and the lower pole will frequently exhibit primary VUR. Finally, large or distally located ureteroceles will sometimes obstruct the bladder outlet, causing severe megacystis and secondary reflux with massive bilateral hydronephrosis.

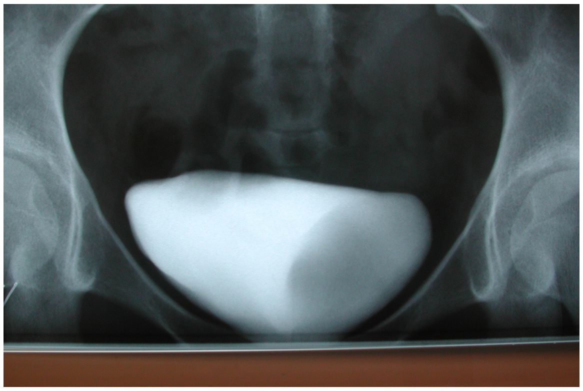

**FIGURE 4.** Ureterocele on VCUG. Filling defect due to a ureterocele.

Studies have demonstrated mixed results with regards to the ability of prenatal diagnosis to improve overall renal function in patients with ureteroceles[1]. However, with the administration of early prophylactic antibiotics, the rate of UTIs has decreased to 3–15% from a historical rate of 70–80%[17,18].

## Posterior Urethral Valves

Posterior urethral valves (PUV) are mucosal flaps that obstruct the outflow of urine to varying degrees. The most common (90%) Type 1 valves have leaflets at the sides of the verumontanum that extend distally to the anterior urethral wall at the level of the urogenital diaphragm[19]. Type 2 valves are very rare and extend proximally from the verumontanum towards the bladder neck. Type 3 valves consist of a diaphragm just distal to the verumontanum with a central perforation; it is thought that these may represent the same entity as Type 1 valves, prior to instrumentation. As the most common cause of newborn bladder outlet obstruction, PUVs occur in approximately 1 in 5,000 to 1 in 8,000 live male births[1]. Approximately 3–9% of prenatal hydronephrosis can be attributed to PUVs [1].

In addition to unilateral or bilateral hydronephrosis from obstruction and secondary reflux, findings on PNUS that are strongly suggestive of PUV include a thick-walled and distended bladder, dilated posterior urethra (making a “keyhole sign”), increased renal echogenicity with subcortical cysts, and oligohydramnios.

PUV is one condition that definitely has improved outcomes with prenatal diagnosis. Detection later in pregnancy might indicate a shorter period of obstruction. Hutton et al. demonstrated that patients with PUV detected after 24 weeks' gestation had a better prognosis than those detected earlier in the pregnancy. Specific second trimester findings that predicted increased postnatal mortality and chronic renal failure include severe upper tract dilation (with APD ≥10 mm) and renal parenchymal changes (increased echogenicity or cystic changes)[20]. In addition, oligohydramnios or anhydramnios with bilateral hydronephrosis is almost universally fatal[1]. Pop-off valves that can relieve upper tract pressure and allow for improved renal development include reflux into a nonfunctioning kidney, a large bladder diverticulum, and urinary ascites – which usually occurs by retroperitoneal renal leak that transudates in to the peritoneal cavity[5].

Prenatal intervention by vesicoamniotic shunt has not been shown definitively to improve postnatal outcome; however, early intervention does appear to decrease postnatal complications, including septicemia and uremia[1].

### Primary Nonrefluxing Megaureter

Megaureter describes a ureteral diameter greater than 1 cm. While this condition can occur secondary to VUR or distal obstruction, it is thought that primary megaureter occurs due to an adynamic portion of the distal ureter. The ureteral dilation can also be due to multiple etiologies, although it is difficult to distinguish these causes prenatally. Aside from the ureteral findings, PNUS can show variable degrees of hydronephrosis.

In general, the natural history of primary megaureter is benign. In a study of 40 infants with primary megaureter, 53% showed complete resolution within 2.9 years, and the rest demonstrated improved or stable findings[21]. Stronger consideration for treatment of primary megaureter is given in cases of bilateral abnormalities or a solitary kidney.

### Other Causes of Prenatal Hydronephrosis: Ectopic Ureter, Ureterovesical Junction Obstruction, Ureteral Stricture

Obstruction at the ureterovesical junction (UVJO) can be due to a narrowing of that segment of ureter or an ectopic ureter. Ectopic ureters terminate outside of the normal trigonal region, and they can be obstructed or nonobstructed. In males, ectopic ureters usually drain to the posterior urethra proximal to the external sphincter. In females, the ureter may insert distally to the bladder neck or into the vagina, causing incontinence. Like ureteroceles, ectopic ureters are associated with duplicated systems, and ultrasonographic findings can include hydroureteronephrosis to the UVJ and signs of associated duplication and reflux. Early detection due to abnormalities on PNUS can lead to decreased morbidity of ectopic ureters, including UTIs and complications of incontinence.

Ureteral stricture anywhere between the UPJ and UVJ can also cause hydronephrosis with ureteral dilation down to the site of obstruction. This is a rare cause of PNUS, and management is usually dictated based on renal function and the severity of obstruction[22].

## GENERAL OUTCOMES OF PRENATAL HYRONEPHROSIS

With regard to prenatal hydronephrosis, the overall benefit of PNUS is debatable. In certain severe cases, including posterior urethral valves with severe obstruction, early intervention can clearly lead to interventions that can prevent mortality and curb morbidity. In other patients, the prenatal finding of hydronephrosis can lead to the early postnatal diagnosis and initiation of antibiotics to prevent complications such as cystitis and pyelonephritis. However, in others, the finding of prenatal hydronephrosis can lead to invasive or unnecessary tests for conditions that would otherwise have had minimal effect on the patients.

In a retrospective study of patients with and without prenatal hydronephrosis, the first group was found to be 12 times more likely to be hospitalized for pyelonephritis[2]. Interestingly, this study did not correlate the number of patients that were on antibiotics; this makes it difficult to deduce the overall effect of detecting hydronephrosis on PNUS.

A meta-analysis of postnatal outcomes found that patients with severe prenatal hydronephrosis have significant risk of postnatal pathology[23]. They created a unique grading system for their analysis to allow for the integration of data from other studies. Notably, they found that 88% of patients with severe prenatal hydronephrosis (second semester APD ≥10 mm) had postnatal pathology; UPJO alone accounted for 54% of patients. Based on these findings, the authors recommended comprehensive investigation of findings in these patients. They reported that further investigations were needed to determine the optimal management of patients found to have mild and moderate prenatal hydronephrosis[23].

## OTHER DIAGNOSTIC TESTS TO CONSIDER

While bilateral hydronephrosis should be carefully followed with sequential fetal ultrasonography, prenatal follow up in the setting of unilateral hydronephrosis is controversial because it would very rarely lead to *in utero* intervention. While not regularly performed, MRI has been useful in some cases in order to clarify anatomy, assess renal parenchymal thickness, or to identify the distal extent of hydroureteronephrosis[22,24].

In cases of oligohydramnios, the karyotype should be assessed and renal function should be evaluated to determine the potential benefit of an intervening procedure. Urine sodium <100 mmol/l, chloride <90 mmol/l, and osmolarity <210 mOsm/l are all findings associated with a good outcome[11]. The fetal urine test can sometimes be of more use when two samples are analyzed and compared.

## FETAL INTERVENTION

Therapeutic fetal intervention to treat hydronephrosis is controversial. Golbus et al. reported on the percutaneous placement of a vesicoamniotic shunt for prune belly syndrome[25]. Currently, fetal vesicoamniotic shunt placement is indicated when oligohydramnios is present in association with bladder outlet obstruction (e.g., posterior urethral valves); in these cases, there is concern for the life of the neonate and the fetus will benefit from *in utero* bladder decompression. A normal karyotype and a singleton pregnancy are also required factors. The technique for fetal vesicoamniotic shunt placement involves bypassing the obstructed urethra by insertion of a double pigtail shunt under ultrasonographic guidance[26]. Quintero et al. described fetal cystoscopy and endoscopic valve ablation, but this technique requires further evaluation[27].

## POSTNATAL EVALUATION OF HYDRONEPHROSIS

Infants diagnosed with prenatal hydronephrosis customarily undergo postnatal imaging evaluation. The timing of postnatal ultrasonography and the indications for VCUG have been controversial. Postnatal ultrasonography to evaluate hydronephrosis is typically delayed for several days due to perinatal dehydration and lower glomerular filtration rate that can potentially lead to false-negative results.

The Society for Fetal Urology proposed the following grading system for ultrasonographic findings of postnatal hydronephrosis[28]:

- Grade 0: Intact central renal complex + normal parenchyma

- Grade 1: Slight central renal complex splitting + normal parenchyma
- Grade 2: Central renal complex splitting confined within renal border + normal parenchyma
- Grade 3: Wide splitting of central renal complex + pelvis dilated outside renal border + calices uniformly dilated + normal parenchyma
- Grade 4: Large dilated calices + further dilation of renal penis + thinning of parenchyma (i.e., less than half that of the contralateral kidney). If bilateral hydronephrosis, then 4 mm is the cutoff used to define parenchymal thinning.

A VCUG is a radiographic test used to detect anomalies such as VUR and ectopic ureteroceles. This test is routinely performed with confirmed postnatal hydronephrosis. However, the use of a VCUG in infants with prenatal hydronephrosis with a normal postnatal ultrasonogram is controversial.

Diuretic renogram is used to evaluate differential renal function and upper tract drainage in children with high-grade hydronephrosis, and suspected UPJO or UVJO. Technicium-99m-diethylene triamine pentaacetic acid ([$^{99m}$Tc] DTPA) and technetium-99m-mercaptoacetyltriglycine ([$^{99m}$Tc] MAG-3) are the two agents commonly used to evaluate for upper tract drainage and function. The rate of decline in radioactivity in the collecting system, i.e., half-life ($t_{1/2}$), is used to evaluate for obstruction: (1) nonobstructive drainage, 0–10 min; (2) indeterminate, 10–20 min; and (3) obstruction, >20 min[29].

## SUMMARY

With the increasingly prevalent use of PNUS, many urologic conditions are detected long before they present with symptoms. The most common urologic finding found on PNUS is hydronephrosis. While the overall benefit of prenatal detection of hydronephrosis is debatable, the finding has a huge effect on fetal interventions, and postnatal studies and management. In this report, we review the definition of prenatal hydronephrosis, the multiple conditions that are associated with this finding, and the potential overall benefit or effect for patients with these conditions.
